# 1,4-Phenyl­ene diallyl bis­(carbonate)

**DOI:** 10.1107/S2414314623001335

**Published:** 2023-02-21

**Authors:** Delia López-Velázquez, Irma Núñez-Méndez, Sylvain Bernès, Isabel Martínez-de la Luz, María Ana Pérez-Cruz

**Affiliations:** a Facultad de Ciencias Químicas, Benemérita Universidad Autónoma de Puebla, 72570 Puebla, Pue., Mexico; b Doctorado en Ciencias Químicas, Facultad de Ciencias Químicas, Benemérita Universidad Autónoma de Puebla, 72570 Puebla, Pue., Mexico; c Instituto de Física, Benemérita Universidad Autónoma de Puebla, Av. San Claudio y 18 Sur, 72570 Puebla, Pue., Mexico; Howard University, USA

**Keywords:** crystal structure, carbonate, allyl group, monomer

## Abstract

The title mol­ecule, C_14_H_14_O_6_, is based on a benzene core di-substituted by allyl carbonate groups in the *para* positions. The mol­ecule is placed on an inversion centre, and the substituents are twisted with respect to the central benzene ring plane. The crystal structure does not include significant inter­molecular inter­actions other than weak C—H⋯O contacts between CH groups in the benzene ring and carbonate O atoms.

## Abstract 2

The title compound represents the first instance of a 1,4-phenyl­ene bis­(carbonate) derivative characterized by X-ray diffraction.

## Graphical abstract

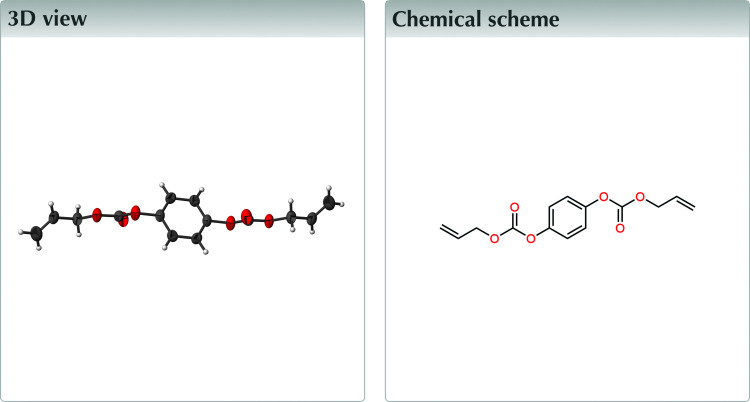

## Structure description

Allylic compounds are common reagents in organic chemistry for obtaining new allyl derivatives and polymeric materials (*e.g*. Nair *et al.*, 2010 ▸). Within this class of compounds, functional allyl aromatic carbonates (Flores Ahuactzin *et al.*, 2009 ▸) are also suitable building blocks to produce diallyl carbonate compounds (López *et al.*, 1997 ▸), as well as reactive homopolycarbonates or copolymers, obtained by free radical polymerization. Concerning diallyl carbonates, they can be used as cross-linking agents (Nair *et al.*, 2010 ▸; López & Burillo, 1991 ▸), and they can also be polymerized to homopolymers or copolymers, such as poly[all­yl(*p*-allyl­carbonate)benzoate] (López-V *et al.*, 2011 ▸) or poly[1-benzoate-2,3-di­allyl­carbonate glycerol] (López *et al.*, 1997 ▸).

The reaction between allyl chloro­formate (ACF) and a diol affords mono allyl carbonate and diallyl carbonate derivatives. The reaction of ACF with hydro­quinone gives allyl-4-hy­droxy­phenyl carbonate (Flores *et al.*, 2009 ▸) and 1,4-phenyl­ene diallyl bis­(carbonate). Herein, we report the structure of the latter. The title compound represents the first instance of a 1,4-phenyl­ene bis­(carbonate) derivative to be characterized by X-ray diffraction.

**Figure 1:**
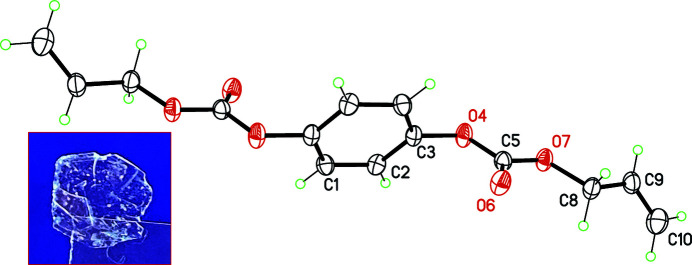
Mol­ecular structure of the title compound. Non-H atoms are drawn at the 30% probability level. Non-labelled atoms are generated by the symmetry operation 1 − *x*, 1 − *y*, −*z*. The inset is the raw material as obtained from the synthesis. The edges of the hexa­gonal flake have dimensions of *ca* 5 mm.

**Figure 2:**
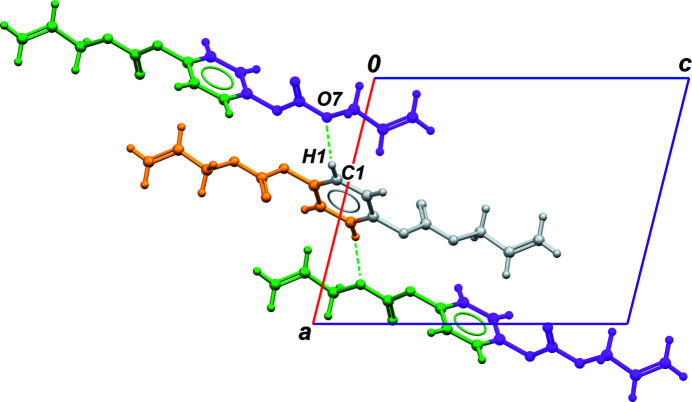
Part of the crystal structure based on C1—H1⋯O7 inter­actions (Table 1 ▸, entry 1). The asymmetric unit is coloured in grey, while orange, green and magenta moieties are generated by inversion, 2_1_ axis and *n* glide plane, respectively.

**Figure 3:**
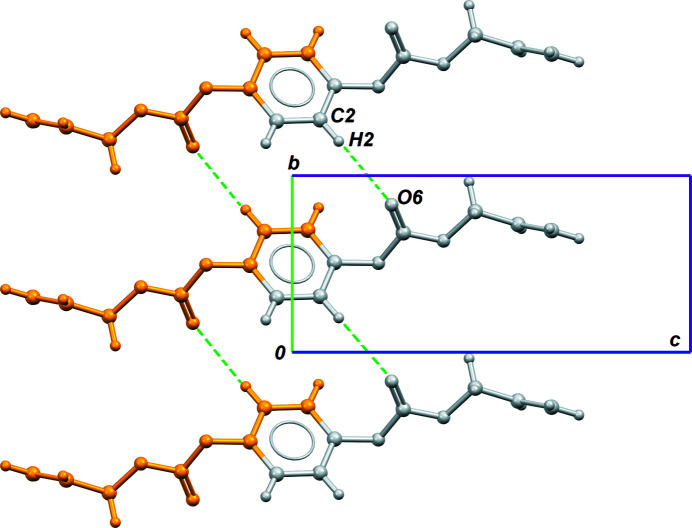
Part of the crystal structure based on C2—H2⋯O6 inter­actions (Table 1 ▸, entry 2). Grey fragments are generated from the asymmetric unit by lattice translations, and orange fragments are generated by inversion.

The mol­ecule lies on an inversion centre in space group *P*2_1_/*n*, with the symmetry element coinciding with the centre of the benzene ring (Fig. 1 ▸). This ring is disubstituted in the *para* positions by allyl carbonate groups, which are not coplanar with the ring: the dihedral angle between the mean plane of the benzene and the plane of the carbonate group O4/C5/O6/O7 is 68.69 (4)°, and the dihedral angle between the carbonate group and the allyl group C8/C9/C10 is 51.1 (2)°. This twisted conformation was previously observed for the four reported X-ray structures bearing a benzene ring substituted by an allyl carbonate group (Flores Ahuactzin *et al.*, 2009 ▸; Herrera-González *et al.*, 2009 ▸; Li *et al.*, 2019 ▸; Schmid *et al.*, 2019 ▸). This conformation does not promote strong inter­molecular contacts in the crystal structure, as hydrogen bonds or π–π inter­actions. The benzene H atoms are, however, engaged in C—H⋯O contacts with neighbouring mol­ecules. The C1—H1 group makes an almost linear contact with the carbonate O atom O7 (Table 1 ▸, entry 1; Fig. 2 ▸), while C2—H2 inter­acts with the carbonyl O atom O6, forming centrosymmetric 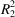 (14) ring motifs in the crystal (Table 1 ▸, entry 2; Fig. 3 ▸).

## Synthesis and crystallization

To a three-neck round-bottom flask connected to an addition funnel, hydro­quinone (2.28 g, 20.7 mmol) was added and dissolved in 20 ml of THF under an argon atmosphere. After continuous agitation, a homogeneous phase was observed in the reaction flask, and NaHCO_3_ (0.86 g, 10.3 mmol), previously dissolved in 5 ml of distilled water, was added. Then, the reaction flask was placed in an ice bath and allyl chloro­formate (1.09 ml, 10.3 mmol) was slowly added dropwise, maintaining the agitation. After complete addition, the reaction was left for 5–10 minutes at 273 K, and then at room temperature for 2 h. After completion of the reaction, the products were extracted in a separation funnel using CH_2_Cl_2_, and dried over anhydrous Na_2_SO_4_. The reaction mixture was filtered and concentrated. The resulting concentrated solution was precipitated into hexane. The precipitate was collected, washed with hexane, and dried *in vacuo* (yield: 1.152 g, 20%). Transparent prismatic single crystals were recovered from this material for X-ray study (see Fig. 1 ▸, inset). ^1^H NMR (500 MHz, CDCl_3_), δ (p.p.m.): 4.75 (*d*, *J* = 5.0 Hz, 4H), 5.34 (*d*, *J* = 10.0 Hz, 2H), 5.44 (*dd*, *J* = 17.5, 1.5 Hz, 2H), 5.90 (*m*, 2H), 7.21 (*s*, 4H); ^13^C NMR (125 MHz, CDCl_3_), δ (p.p.m.): 69.3 (–CH_2_–), 119.7 (=CH_2_), 122.0 (benzene), 131.1 (=CH), 148.6 (benzene), 154.3 (C=O); FTIR (ATR, ν, cm^−1^): 3082 (C*sp* ^2^—H), 2960 (C*sp* ^3^—H), 1757 (C=O), 1649 (C=C, all­yl), 1602 (C=C, aromatic), 770 (aromatic ring), 730 (C*sp* ^3^—H).

## Refinement

Crystal data, data collection and structure refinement details are summarized in Table 2 ▸.

## Supplementary Material

Crystal structure: contains datablock(s) I, global. DOI: 10.1107/S2414314623001335/bv4045sup1.cif

Structure factors: contains datablock(s) I. DOI: 10.1107/S2414314623001335/bv4045Isup2.hkl

Supporting information file. DOI: 10.1107/S2414314623001335/bv4045Isup3.cml

CCDC reference: 2241801

Additional supporting information:  crystallographic information; 3D view; checkCIF report

## Figures and Tables

**Table 1:** Hydrogen-bond geometry (Å, °)

*D*—H⋯*A*	*D*—H	H⋯*A*	*D*⋯*A*	*D*—H⋯*A*
C1—H1⋯O7^i^	0.93	2.59	3.5100 (16)	169
C2—H2⋯O6^ii^	0.93	2.57	3.4555 (16)	160

Symmetry codes: (i) 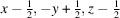 ; (ii) 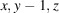 .

**Table 2:** Experimental details

Crystal data	
Chemical formula	C_14_H_14_O_6_
*M*_r_	278.25
Crystal system, space group	Monoclinic, *P*2_1_/*n*
Temperature (K)	253
*a*, *b*, *c* (Å)	10.2808 (13), 5.4764 (6), 12.7396 (15)
β (°)	104.070 (9)
*V* (Å^3^)	695.74 (14)
*Z*	2
Radiation type	Ag *K*α, λ = 0.56083 Å
μ (mm^−1^)	0.06
Crystal size (mm)	0.60 × 0.50 × 0.50

Data collection	
Diffractometer	Stoe Stadivari
Absorption correction	Multi-scan (*X-AREA*; Stoe & Cie, 2018 ▸)
*T*_min_, *T*_max_	0.536, 1.000
No. of measured, independent and observed [*I* > 2σ(*I*)] reflections	16744, 1620, 1305
*R*_int_	0.029
(sin θ/λ)_max_ (Å^−1^)	0.653

Refinement	
*R*[*F* ^2^ > 2σ(*F* ^2^)], *wR*(*F* ^2^), *S*	0.035, 0.102, 1.04
No. of reflections	1620
No. of parameters	98
H-atom treatment	H atoms treated by a mixture of independent and constrained refinement
Δρ_max_, Δρ_min_ (e Å^−3^)	0.18, −0.15

Computer programs: *X-AREA* (Stoe & Cie, 2018 ▸), *SHELXT2018/2* (Sheldrick, 2015*a* ▸), *SHELXL2018/3* (Sheldrick, 2015*b* ▸), *XP* in *SHELXTL-Plus* (Sheldrick, 2008 ▸), *Mercury* (Macrae *et al.*, 2020 ▸) and *publCIF* (Westrip, 2010 ▸).

## full crystallographic data

### Crystal data

**Table:**

C_14_H_14_O_6_	*F*(000) = 292
*M**_r_* = 278.25	*D*_x_ = 1.328 Mg m^−^^3^
Monoclinic, *P*2_1_/*n*	Ag *K*α radiation, λ = 0.56083 Å
*a* = 10.2808 (13) Å	Cell parameters from 19549 reflections
*b* = 5.4764 (6) Å	θ = 2.3–31.6°
*c* = 12.7396 (15) Å	µ = 0.06 mm^−^^1^
β = 104.070 (9)°	*T* = 253 K
*V* = 695.74 (14) Å^3^	Prism, colourless
*Z* = 2	0.60 × 0.50 × 0.50 mm

### Data collection

**Table:**

Stoe Stadivari diffractometer	1620 independent reflections
Radiation source: Sealed X-ray tube, Axo Astix-f Microfocus source	1305 reflections with *I* > 2σ(*I*)
Graded multilayer mirror monochromator	*R*_int_ = 0.029
Detector resolution: 5.81 pixels mm^-1^	θ_max_ = 21.5°, θ_min_ = 2.3°
ω scans	*h* = −13→13
Absorption correction: multi-scan (X-AREA; Stoe & Cie, 2018)	*k* = −7→7
*T*_min_ = 0.536, *T*_max_ = 1.000	*l* = −16→16
16744 measured reflections	

### Refinement

**Table:**

Refinement on *F*^2^	Secondary atom site location: difference Fourier map
Least-squares matrix: full	Hydrogen site location: mixed
*R*[*F*^2^ > 2σ(*F*^2^)] = 0.035	H atoms treated by a mixture of independent and constrained refinement
*wR*(*F*^2^) = 0.102	*w* = 1/[σ^2^(*F*_o_^2^) + (0.0441*P*)^2^ + 0.1879*P*] where *P* = (*F*_o_^2^ + 2*F*_c_^2^)/3
*S* = 1.04	(Δ/σ)_max_ < 0.001
1620 reflections	Δρ_max_ = 0.18 e Å^−^^3^
98 parameters	Δρ_min_ = −0.15 e Å^−^^3^
0 restraints	Extinction correction: SHELXL-2018/3 (Sheldrick 2015b), Fc^*^=kFc[1+0.001xFc^2^λ^3^/sin(2θ)]^-1/4^
0 constraints	Extinction coefficient: 0.023 (6)
Primary atom site location: dual	

### Special details

**Table:**

Refinement. Allyl H atoms (H10*a* and H10*b*) were refined with free coordinates, and other H atoms were placed on calculated positions. All H atoms were refined with isotropic displacements, calculated as *U*_iso_(H) = 1.2×*U*_eq_(carrier C atom).

### Fractional atomic coordinates and isotropic or equivalent isotropic displacement parameters (Å^2^)

**Table:**

	*x*	*y*	*z*	*U*_iso_*/*U*_eq_	
C1	0.41290 (13)	0.3086 (2)	−0.03895 (10)	0.0404 (3)	
H1	0.354051	0.182011	−0.066169	0.049*	
C2	0.47808 (13)	0.3163 (2)	0.06961 (10)	0.0401 (3)	
H2	0.464542	0.194551	0.116732	0.048*	
C3	0.56326 (12)	0.5078 (2)	0.10622 (9)	0.0365 (3)	
O4	0.63189 (9)	0.50484 (17)	0.21618 (7)	0.0450 (3)	
C5	0.59396 (12)	0.6762 (2)	0.27902 (9)	0.0366 (3)	
O6	0.51476 (10)	0.83464 (18)	0.24966 (7)	0.0498 (3)	
O7	0.66007 (9)	0.63120 (18)	0.37984 (6)	0.0440 (3)	
C8	0.62690 (13)	0.7968 (3)	0.45975 (10)	0.0444 (3)	
H8A	0.532199	0.785459	0.457866	0.053*	
H8B	0.646954	0.964013	0.444160	0.053*	
C9	0.70837 (14)	0.7236 (3)	0.56723 (10)	0.0476 (3)	
H9	0.799945	0.700847	0.575414	0.057*	
C10	0.6593 (2)	0.6888 (3)	0.65149 (13)	0.0630 (4)	
H10A	0.7141 (18)	0.644 (4)	0.7198 (16)	0.076*	
H10B	0.5653 (19)	0.700 (4)	0.6461 (14)	0.076*	

### Atomic displacement parameters (Å^2^)

**Table:**

	*U*^11^	*U*^22^	*U*^33^	*U*^12^	*U*^13^	*U*^23^
C1	0.0448 (6)	0.0346 (6)	0.0399 (7)	−0.0038 (5)	0.0063 (5)	−0.0041 (5)
C2	0.0516 (7)	0.0331 (6)	0.0356 (6)	0.0008 (5)	0.0109 (5)	0.0029 (5)
C3	0.0409 (6)	0.0367 (6)	0.0290 (6)	0.0076 (5)	0.0031 (4)	−0.0027 (5)
O4	0.0532 (5)	0.0449 (5)	0.0309 (4)	0.0150 (4)	−0.0012 (4)	−0.0038 (4)
C5	0.0368 (6)	0.0387 (6)	0.0320 (6)	0.0025 (5)	0.0040 (4)	0.0004 (5)
O6	0.0567 (6)	0.0520 (6)	0.0366 (5)	0.0203 (4)	0.0035 (4)	0.0010 (4)
O7	0.0472 (5)	0.0508 (5)	0.0291 (4)	0.0135 (4)	0.0000 (3)	−0.0038 (4)
C8	0.0470 (7)	0.0497 (7)	0.0343 (6)	0.0064 (6)	0.0057 (5)	−0.0070 (5)
C9	0.0469 (7)	0.0544 (8)	0.0371 (7)	0.0016 (6)	0.0019 (5)	−0.0077 (6)
C10	0.0770 (11)	0.0669 (10)	0.0434 (8)	0.0006 (9)	0.0112 (7)	0.0051 (7)

### Geometric parameters (Å, º)

**Table:**

C1—C3^i^	1.3811 (17)	O7—C8	1.4640 (15)
C1—C2	1.3830 (17)	C8—C9	1.4763 (17)
C1—H1	0.9300	C8—H8A	0.9700
C2—C3	1.3733 (18)	C8—H8B	0.9700
C2—H2	0.9300	C9—C10	1.306 (2)
C3—O4	1.4071 (14)	C9—H9	0.9300
O4—C5	1.3513 (14)	C10—H10A	0.947 (19)
C5—O6	1.1863 (14)	C10—H10B	0.954 (18)
C5—O7	1.3221 (14)		
			
C3^i^—C1—C2	118.84 (11)	C5—O7—C8	114.10 (9)
C3^i^—C1—H1	120.6	O7—C8—C9	107.53 (10)
C2—C1—H1	120.6	O7—C8—H8A	110.2
C3—C2—C1	118.48 (11)	C9—C8—H8A	110.2
C3—C2—H2	120.8	O7—C8—H8B	110.2
C1—C2—H2	120.8	C9—C8—H8B	110.2
C2—C3—C1^i^	122.68 (11)	H8A—C8—H8B	108.5
C2—C3—O4	116.95 (11)	C10—C9—C8	123.81 (14)
C1^i^—C3—O4	120.30 (11)	C10—C9—H9	118.1
C5—O4—C3	115.77 (9)	C8—C9—H9	118.1
O6—C5—O7	126.45 (11)	C9—C10—H10A	122.1 (12)
O6—C5—O4	126.58 (11)	C9—C10—H10B	121.3 (11)
O7—C5—O4	106.96 (9)	H10A—C10—H10B	116.6 (16)
			
C3^i^—C1—C2—C3	0.5 (2)	C3—O4—C5—O7	174.50 (10)
C1—C2—C3—C1^i^	−0.5 (2)	O6—C5—O7—C8	0.88 (19)
C1—C2—C3—O4	−177.58 (11)	O4—C5—O7—C8	−177.99 (10)
C2—C3—O4—C5	−110.62 (12)	C5—O7—C8—C9	−179.91 (11)
C1^i^—C3—O4—C5	72.25 (15)	O7—C8—C9—C10	−130.59 (16)
C3—O4—C5—O6	−4.37 (19)		

Symmetry code: (i) −*x*+1, −*y*+1, −*z*.

### Hydrogen-bond geometry (Å, º)

**Table:**

*D*—H···*A*	*D*—H	H···*A*	*D*···*A*	*D*—H···*A*
C1—H1···O7^ii^	0.93	2.59	3.5100 (16)	169
C2—H2···O6^iii^	0.93	2.57	3.4555 (16)	160

Symmetry codes: (ii) *x*−1/2, −*y*+1/2, *z*−1/2; (iii) *x*, *y*−1, *z*.

## References

[bb1] Flores Ahuactzin, V. H., López, D. & Bernès, S. (2009). *Acta Cryst.* E**65**, o1603. 10.1107/S1600536809022387 PMC2969403 21582877

[bb2] Herrera-González, A. M., López-Velázquez, D. & Bernès, S. (2009). *Acta Cryst.* E**65**, o2810–o2811. 10.1107/S1600536809041142 PMC2971017 21578402

[bb3] Li, M., Chen, H., Zheng, K., Liu, X., Xiao, S. & Zhang, N. (2019). *Inorg. Chim. Acta*, **495**, 119000.

[bb4] López, D. & Burillo, G. (1991). *ACS Symp. Ser.* **475**, 262–270.

[bb5] López, D., Plata, P., Burillo, G. & Medina, C. (1997). *Radiat. Phys. Chem.* **50**, 171–173.

[bb6] López-V, D., Herrera-G, A. M. & Castillo-Rojas, S. (2011). *Radiat. Phys. Chem.* **80**, 481–486.

[bb7] Macrae, C. F., Sovago, I., Cottrell, S. J., Galek, P. T. A., McCabe, P., Pidcock, E., Platings, M., Shields, G. P., Stevens, J. S., Towler, M. & Wood, P. A. (2020). *J. Appl. Cryst.* **53**, 226–235. 10.1107/S1600576719014092 PMC6998782 32047413

[bb8] Nair, D. P., Cramer, N. B., Scott, T. F., Bowman, C. N. & Shandas, R. (2010). *Polymer*, **51**, 4383–4389. 10.1016/j.polymer.2010.07.027 PMC2975462 21072253

[bb9] Schmid, M., Grossmann, A. S., Mayer, P., Müller, T. & Magauer, T. (2019). *Tetrahedron*, **75**, 3195–3215. 10.1016/j.tet.2019.03.010 PMC6544523 31160829

[bb10] Sheldrick, G. M. (2008). *Acta Cryst.* A**64**, 112–122. 10.1107/S0108767307043930 18156677

[bb11] Sheldrick, G. M. (2015*a*). *Acta Cryst.* A**71**, 3–8.

[bb12] Sheldrick, G. M. (2015*b*). *Acta Cryst.* C**71**, 3–8.

[bb13] Stoe & Cie (2018). *X-AREA* and *X-RED32*, Stoe & Cie, Darmstadt, Germany.

[bb14] Westrip, S. P. (2010). *J. Appl. Cryst.* **43**, 920–925.

